# Patient-reported outcomes as prognostic indicators for overall survival and progression-free survival in brain tumor patients – a systematic review and meta-analysis of randomized clinical trials

**DOI:** 10.1007/s11060-025-05419-w

**Published:** 2026-01-05

**Authors:** Sai Sanikommu, Alejandro N. Santos, Christoph Wipplinger, Bashar Dawoud, Ricardo J. Komotar, Victor M. Lu

**Affiliations:** 1 Department of Neurosurgery, Miller School of Medicine, University of Miami, Miami, FL 33136 USA; 2 Department of Neurosurgery, Cleveland Clinic, Weston, FL USA

**Keywords:** Patient-reported outcomes, Brain tumors, Glioblastoma, Randomized clinical trials, Prognosis, Systematic review, Meta-analysis

## Abstract

**Background:**

Patient-reported outcomes (PROs) are increasingly recognized as essential endpoints in neuro-oncology, yet their prognostic value for survival across brain tumor trials remains incompletely defined. We conducted a systematic review and meta-analysis to quantify the association between key PRO domains and overall survival (OS) and progression-free survival (PFS)in patients with glioma.

**Methods:**

Following PRISMA 2020 guidelines, we included randomized controlled trials that reported baseline PROs using the European Organisation for the Research and Treatment of Cancer Quality of Life Questionnaire Core 30 or European Organisation for Research and Treatment of Cancer Quality of Life Questionnaire-Brain Neoplasm-20 questionnaires, with corresponding survival outcomes. Hazard ratios (HRs) per 10-point increase in each PRO domain were extracted. Random-effects models generated pooled HRs. Heterogeneity, risk of bias (ROB 2), and certainty of evidence (GRADE) were assessed.

**Results:**

Eight trials comprising 6,846 patients were included. Higher baseline cognitive functioning was significantly associated with improved OS (HR = 0.94, 95% CI [0.91–0.97]), as was physical functioning (HR = 0.97, 95% CI [0.94-1.00]). Pooled functional domains showed a protective association (HR = 0.96, 95% CI [0.93-1.00]), while BN-20 domains showed no association with overall survival. Pooled analysis of studies reporting EORTC QLQ-C30 scales was associated with improved PFS (HR = 0.99, 95% CI [0.99–0.99]). Subgroup analysis of physical functioning showed an association with improved PFS (HR = 0.99, 95% CI [0.97-1.0]), and the pooled analysis of all functional scales showed the same direction (HR = 0.99, 95% CI [0.99–0.99]).

**Conclusions:**

Functional and cognitive PRO domains appear to have potential to be robust prognostic markers of survival in glioma trials, and these findings support the complementary role of PROs alongside clinical, radiographic, and molecular measures. Our findings support integrating PROs into response assessment selection in future neuro-oncology trials.

**Supplementary Information:**

The online version contains supplementary material available at 10.1007/s11060-025-05419-w.

## Introduction

Quality of life (QoL) and functional status are pivotal aspects in the management of patients with brain tumors [1, 2]. Nonetheless, prognostic evaluation in neuro-oncology primarily relies on objective clinical and molecular markers, including age, performance status (an objective assessment by physician), extent of resection, IDH mutation status, and MGMT promoter methylation [3–6]. Although patient reported QoL metrics are systematically collected in clinical trials using validated instruments such as the European Organisation for the Research and Treatment of Cancer Quality of Life Questionnaire Core 30 (EORTC QLQ C30) [7] and European Organisation for Research and Treatment of Cancer Quality of Life Questionnaire-Brain Neoplasm-20 (EORTC QLQ BN20) [8], their use predominantly serves as secondary endpoints to assess treatment tolerability rather than as prognostic factors for survival [8–10].

In contemporary neuro-oncology, clinical trial endpoints and treatment response assessments are primarily defined by the Response Assessment in Neuro-Oncology (RANO) criteria [11]. RANO integrates radiographic changes with selected neurological and corticosteroid-related clinical features to determine progression-free survival (PFS) and overall survival (OS) [11]. Yet RANO excludes patient-reported QoL measures, despite the accumulating evidence indicating that impairments in baseline physical function, cognitive ability, fatigue, and overall health perception may be linked to poorer survival outcomes in patients with gliomas and brain metastases [2, 12–20]. Multiple randomized controlled trials (RCTs) suggest that baseline QoL can serve as an independent predictor of OS and PFS in neuro-oncological patients [13–20]. Nonetheless, these findings have yet to be unified, often constrained by small sample sizes, heterogeneous QoL measurement instruments, and variations in analytical methodologies. Importantly, to date, no systematic review or meta-analysis has consolidated evidence across studies or quantified the prognostic significance of various QoL domains in brain tumors relative to conventional clinical and molecular prognostic factors.

Therefore, we conducted the first systematic review and meta-analysis to evaluate whether baseline patient reported QoL may be an independent prognostic factor for survival in patients with brain tumors. Specifically, we aimed to quantify the pooled hazard ratio (HR) for OS and PFS associated with each QoL domain and compare the prognostic strength of functional, symptom, and global QoL scales.

## Methods

This systematic review and meta-analysis were conducted in accordance with the Preferred Reporting Items for Systematic Reviews and Meta-Analyses (PRISMA) 2020 guidelines. The protocol was prospectively registered in the PROSPERO international prospective register of systematic reviews (registration number: 1248134).

Data from this study is available upon reasonable request from the corresponding author.

### Search strategy and eligibility criteria

We conducted a systematic literature search of PubMed (MEDLINE), Embase, and the Scopus from January 2000 to November 2025 (dates were selected based as the present day EORTC QLQ-C30 tool was introduced in 1997), to identify randomized controlled trials (RCTs) evaluating the prognostic significance of baseline health-related quality of life (HRQoL) and functional patient-reported outcomes (PROs) in adults with primary or metastatic brain tumors. The complete search strategy is reported in Supplementary Table S1. Eligible studies met the following criteria: (1) enrolled adult patients (≥ 18 years) in prospective phase II-IV RCTs investigating any therapeutic modality for brain tumors; (2) included at least one validated PRO or HRQoL instrument assessed at baseline (EORTC QLQ-C30/BN20); (3) reported OS and/or PFS outcomes; and (4) performed at least one univariate or multivariate survival analysis assessing the association between baseline PROs and survival while adjusting for established clinical prognostic factors such as age, objective performance status (KPS/WHO PS), extent of resection, MGMT methylation status, steroid use, or treatment arm. Studies lacking survival analyses, non-RCT designs, pediatric populations, or lacking extractable hazard ratios were excluded. Detailed inclusion and exclusion criteria are summarized in Supplementary Table S2.

### Study selection and data collection

**Fig. 1:**
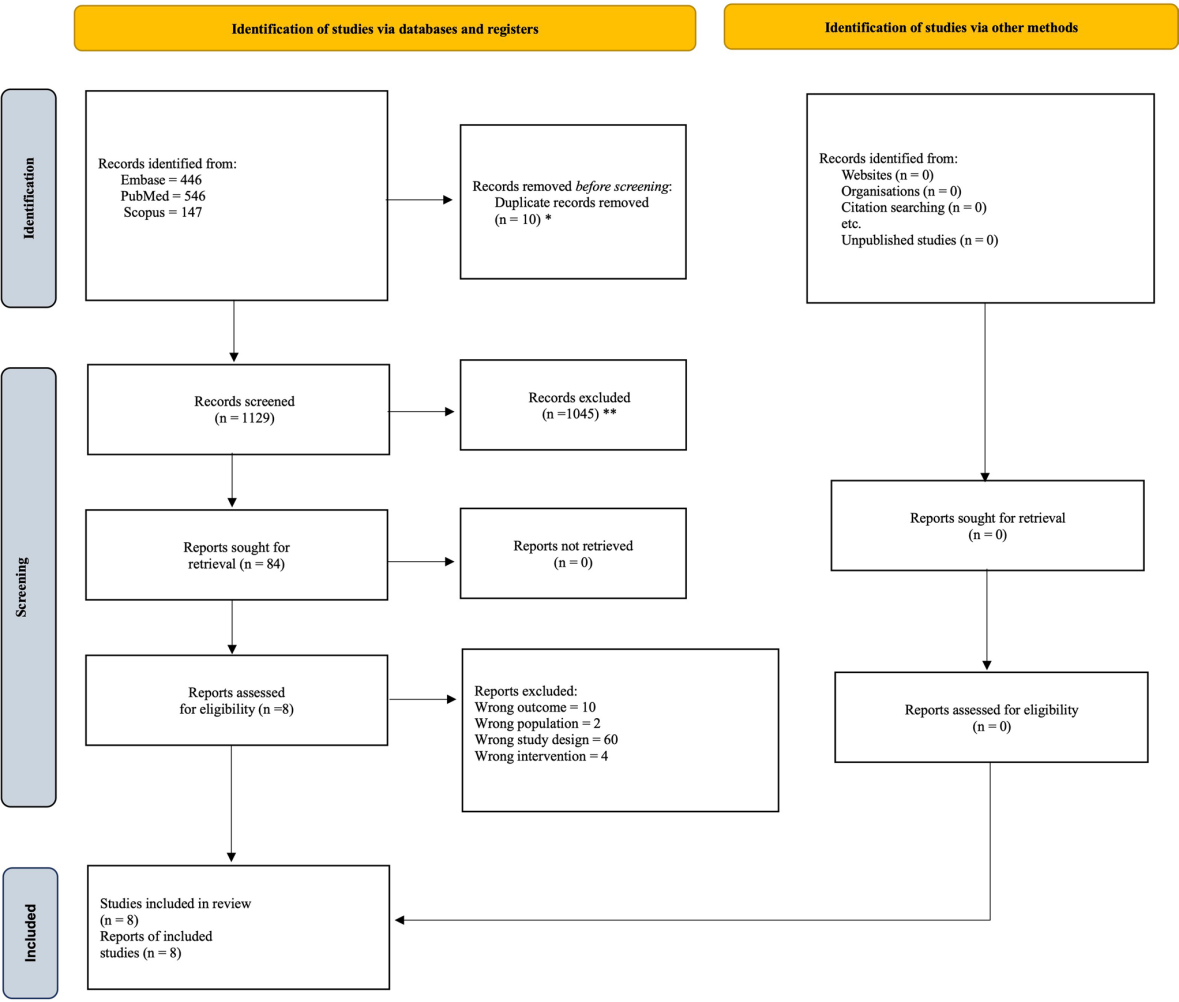
PRISMA flowchart of study selection. * With automation tool; ** Without automation tool

Study selection and screening were conducted in accordance with PRISMA 2020 guidelines. All records retrieved from the database search were imported into EndNote X9 for initial management, where duplicate entries were identified and removed. The deduplicated dataset was then transferred to Mendeley and exported in RIS format for systematic screening using the Rayyan platform (https://www.rayyan.ai/). Two independent reviewers (S.S. and B.D.) screened titles and abstracts, followed by full-text assessment of all studies that met preliminary eligibility criteria. Discrepancies at any stage were resolved through consensus, with two senior investigators (A.N.S. and V.M.L.) serving as adjudicators when necessary. To ensure completeness, reference lists of all included studies and relevant reviews were manually examined. The selection process is summarized in the PRISMA flow diagram (Fig. 1).

For inclusion in the meta-analysis component of this study, additional prespecified criteria were applied. Eligible randomized controlled trials were required to report HRs derived from multivariable Cox proportional hazards models assessing the association between baseline PROs or functional status measures and OS or PFS. Only analyses that adjusted for relevant clinical covariates (e.g., age, extent of resection, MGMT methylation, performance status) were considered, ensuring that the prognostic contribution of PROs was evaluated independently of established clinical predictors. For secondary or pooled analyses derived from multiple randomized controlled trials, included populations were carefully cross-checked against other eligible studies to assess potential overlap. When overlap was identified, data were included only once, prioritizing the most comprehensive multivariable-adjusted analysis to avoid double-counting of patient populations.

**Fig. 2:**
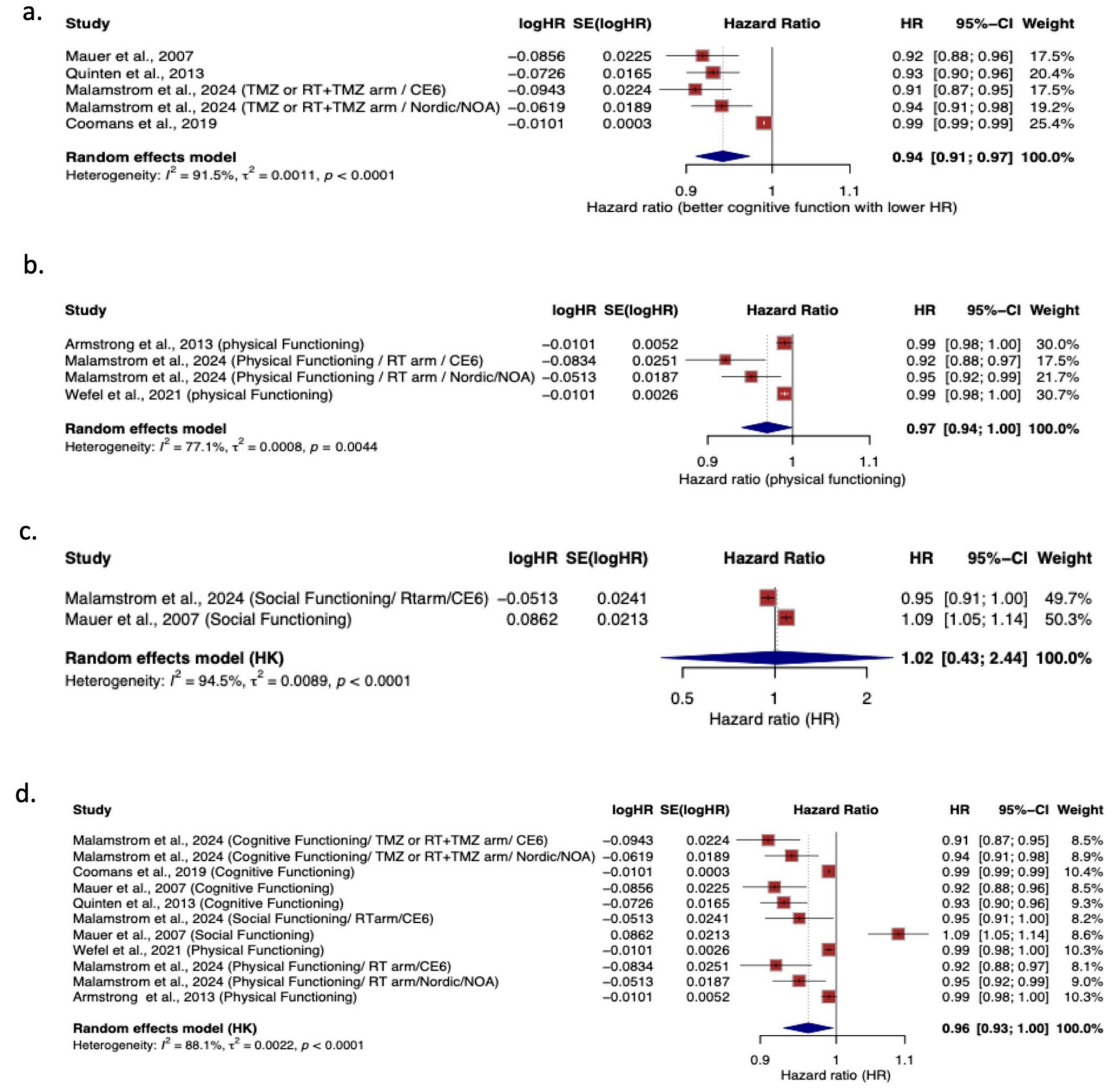
Association between European organisation for research and treatment of cancer quality of life questionnaire core 30, Domains and overall survival. (**a**) cognitive functioning, (**b**) physical functioning, (**c**) social functioning, and (**d**) combined functional domains. Reported as hazard ratios (HRs) with 95% CIs. HR <1 indicates improved survival with higher functioning scores

**Fig. 3:**
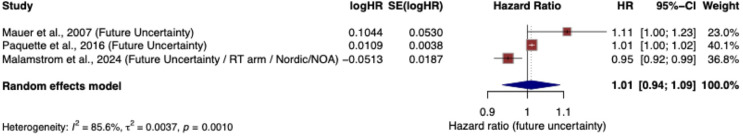
Association between European organisation for the research and treatment of cancer quality of life questionnaire brain neoplasm 20 domain, future uncertainty, and overall survival. with 95% CIs. HR <1 indicates improved survival with higher functioning scores

**Fig. 4:**
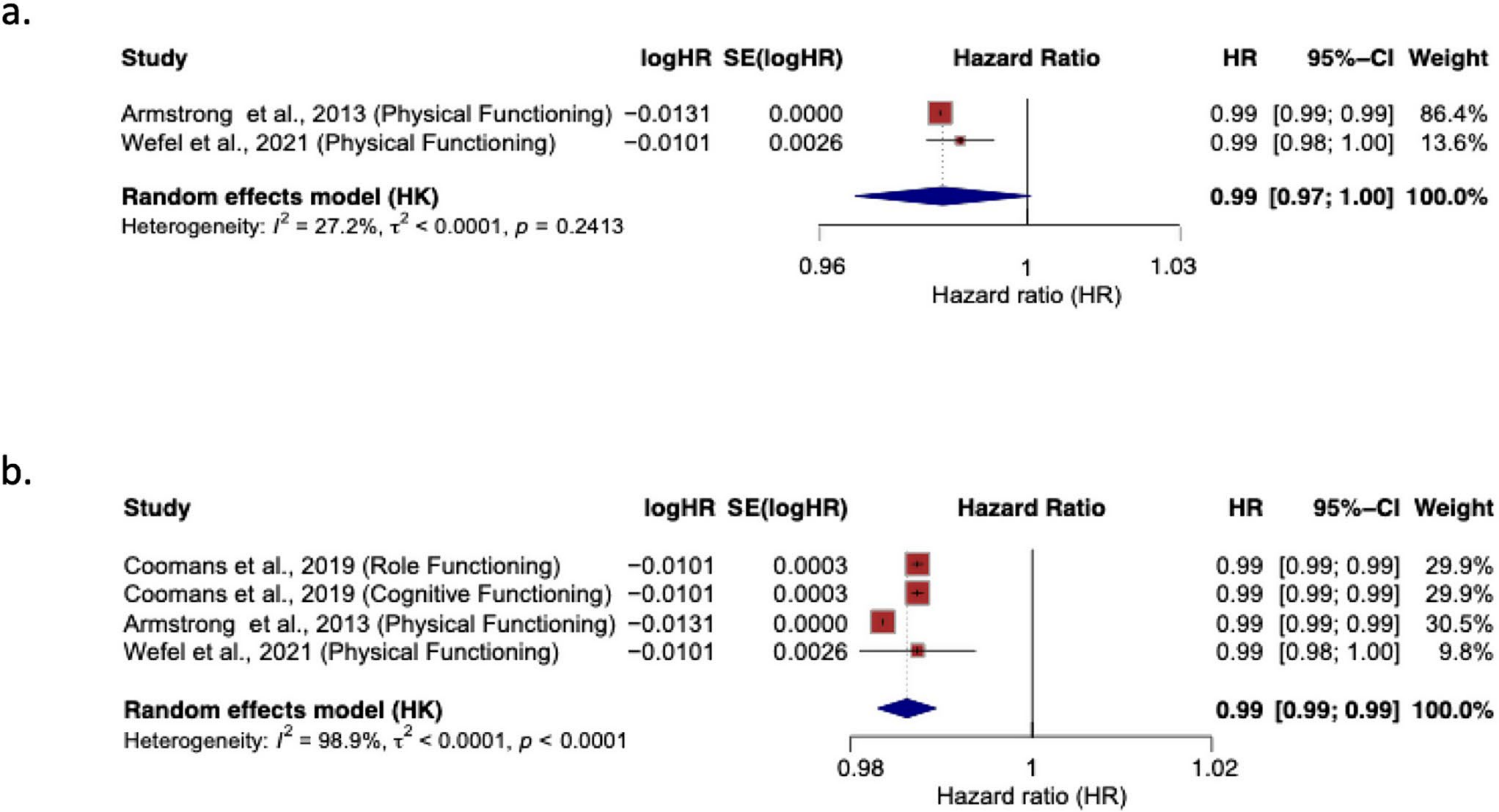
Association between European organisation for the research and treatment of cancer quality of life core 30 domains and overall survival. (**a**) physical functioning, (**b**) combined functional domains. Reported as hazard ratios (HRs) with 95% CIs. HR < 1 indicates improved survival with higher functional scores

Because no unified PRO instrument exists for neuro-oncology trials, studies using the EORTC QLQ-C30/BN20 were eligible, provided that PRO domains were reported as continuous variables. Continuous PRO reporting was required to maintain methodological consistency and to enable appropriate pooling of HRs across trials. When multiple publications originated from overlapping trial populations, only the most complete and methodologically robust dataset was retained to avoid duplication. All data used in the meta-analysis were extracted from published sources.

The EORTC QLQ-C30 is a well-validated and widely used PRO instrument in oncology that assesses multiple domains of health-related quality of life in patients with cancer. It includes five functional scales (physical, role, emotional, cognitive, and social), three symptom scales (fatigue, pain, and nausea/vomiting), a global health status/quality-of-life scale, and several single-item measures assessing additional symptoms such as appetite loss, insomnia, constipation, and diarrhea [7]. The EORTC QLQ-BN20 is a validated, brain tumor–specific patient-reported outcome module designed to complement the QLQ-C30 by capturing symptoms and functional impairments unique to patients with primary or metastatic brain tumors. It comprises multiple symptom domains, including future uncertainty, visual disorders, motor dysfunction, and communication deficits, as well as additional items assessing headaches, seizures, drowsiness, hair loss, and treatment-related concerns. This instrument enables a comprehensive evaluation of neurologic and tumor-related quality-of-life burdens in neuro-oncology populations [8].

### Risk of bias and certainty assessment

The methodological quality of the included studies was assessed in accordance with the Cochrane Handbook for Systematic Reviews of Interventions [21]. Two independent reviewers (S.S. and A.N.S.) applied the Risk of Bias tool for Randomized trials (ROB 2) tool to evaluate potential bias across domains [22]. The certainty of evidence supporting pooled estimates was graded using the Grading of Recommendations, Assessment, Development, and Evaluation (GRADE) framework, considering risk of bias, consistency, directness, precision, and publication bias [21].

### Statistical analysis

Descriptive statistics were used to summarize the characteristics of the included studies and their patient populations. In the systematic review component, all PRO domains demonstrating significant associations with OS in the original analyses were qualitatively synthesized. For the quantitative meta-analysis, only studies that reported multivariable HRs for continuous baseline scores of the EORTC QLQ-C30 or EORTC QLQ BN-20 instrument were eligible for pooling, as this measure was the most consistently applied and methodologically comparable across trials. For each study, log hazard ratios (logHRs) and their corresponding standard errors were calculated from the reported HRs and 95% confidence intervals. Pooled effect estimates were generated using a random-effects model with Hartung-Knapp adjustment, implemented through the meta package in R, applying inverse-variance weighting to account for between-study heterogeneity. Statistical heterogeneity was quantified using the I² statistic, Cochran’s Q test, and the estimated between-study variance (τ²). Analyses were conducted for each EORTC QLQ-C30 and EORTC QLQ-BN20 domain when two or more studies reported comparable continuous estimates, and additional stratified analyses were performed across functional domains to evaluate broader patterns of prognostic relevance. Given the anticipated clinical and methodological heterogeneity across studies, pooled analyses were restricted to PRO domains reported by multiple studies with comparable definitions and adjustment strategies. Where fewer than three studies contributed to a domain or heterogeneity was substantial, results were interpreted cautiously and supplemented with narrative synthesis. All statistical tests were two-sided with a significance threshold of *P* < 0.05. All analyses, including forest plot generation and heterogeneity assessment, were performed using R version 4.3.2.

## Results

### Study characteristics

Following an initial screening of the titles and abstracts of 1,139 studies, a total of 84 full-text articles were evaluated for eligibility. This process culminated in the inclusion of 6846 patients across eight RCTs [13–20]. Of these, two studies by Coomans et al.^15^, and Quinten et al.^18^, analyzed individual patient-level data derived from 15 to 2 RCTs, respectively. The constituent trials were cross-referenced with other included studies and overlapping trial-level publications were not entered separately into the meta-analysis to preserve independence of patient populations. There were 6 (75%) studies were conducted in Europe, while 2 (25%) in the USA. Six (75%) studies analysed glioblastoma only [13–15, 17, 19, 20], while 1 (12.5%) study reported oligodendroglioma outcomes [16], and the study by Quinten et al.^18^ did not specify brain tumor type. There were 7 (87.5%) studies who used both the EORTC QLQ-C30 and EORTC QLQ BN20, and 1 (12.5%) study used EORTC QLQ-C30 as PRO instruments to assess QoL among patients [13–20]. All the studies included in this systematic review and meta-analysis found at least 1 PRO measure to be significantly associated with predicting OS and PFS. A comprehensive summary of the significant variables identified in multivariable analyses for each study is presented in Table 1.

**Table 1:** Summary of included randomized controlled trials evaluating pros and their association with OS and PFS

First Author (year)	Trail Phase	Country	Cancer type	Sample Size	PRO Measure Analyzed in Study	OS/PFS	Significant PRO Measures on Multivariable Analysis	Covariates Adjusted in the Multivariable Analysis
Coomans et al., 2019	II/III	EU	Glioma	3211	QLQ-C30	OS	Role Functioning, Cognitive Functioning	Age; sex; WHO performance status; tumor type (WHO grade); prior resection (yes/no); treatment category; stratified by disease stage (newly diagnosed vs. recurrent).
					QLQ-BN20	OS	Motor Dysfunction	Age; sex; WHO performance status; tumor type (WHO grade); prior resection (yes/no); treatment category; stratified by disease stage (newly diagnosed vs. recurrent).
					QLQ-C30	PFS	Role Functioning, Cognitive Functioning, Nausea and Vomiting, Appetite Loss	Age; sex; WHO performance status; tumor type (WHO grade); prior resection (yes/no); treatment category; stratified by disease stage (newly diagnosed vs. recurrent).
Paquette et al., 2016	II	France	Glioblastoma	102	QLQ-BN20	OS	Future Uncertainty	independently selected clinical, biological, and tumoral prognostic factors identified via multivariable Cox modeling; HRQoL domains (QLQ-C30 and QLQ-BN20) selected using stepwise procedures.
Quinten et al., 2013	II/III	EU	Unkonwn	829	QLQ-C30	OS	Cognitive Functioning	Age; sex; WHO performance status; stratified by trial (and distant metastasis where reported). *
Armstrong et al., 2013	III	USA	Glioblastoma	182	QLQ-C30	OS	Physical Functioning, Nausea and Vomiting	RPA class; MGMT promoter methylation status (with stepwise-selected NCB/PRO predictors)^$^
					QLQ-C30	PFS	Physical Functioning, Constipation	RPA class; MGMT promoter methylation status (with stepwise-selected NCB/PRO predictors)^$^
Mauer et al., 2007	III	Europe/Canada	Glioblastoma	490	QLQ-C30	OS	Global Health Status, Cognitive Functioning, Social Functioning	Age; performance status; extent of surgery; baseline corticosteroid use; MMSE score; stratified by MGMT promoter methylation status and treatment arm.
Mauer et al., 2007	III	Europe	Anaplastic Oligodendrogliomas	247	QLQ-C30	OS	Emotional Functioning	Age; sex; WHO performance status; extent of surgery; tumor histology; treatment arm
					QLQ-BN20	OS	Future Uncertainty, Communication Deficit, Weakness of Legs	Age; sex; WHO performance status; extent of surgery; tumor histology; treatment arm
Wefel et al., 2021	III	USA	Glioblastoma	508	QLQ-C30	OS	Physical Functioning	RPA class and MGMT promoter methylation status (unmethylated vs. methylated)
					QLQ-BN20	OS	Communication Deficit	RPA class and MGMT promoter methylation status (unmethylated vs. methylated)
					QLQ-C30	PFS	Physical Functioning	RPA class and MGMT promoter methylation status (unmethylated vs. methylated)
Malamstrom et al., 2024	III	Europe/Canada	Glioblastoma	281 (RT arm/CE6)	QLQ-C30	OS	Social Functioning, Physical Functioning,	age; sex; WHO performance status; surgery type; baseline steroid use; MGMT status.
					QLQ-BN20	OS	Visual Disorder	age; sex; WHO performance status; surgery type; baseline steroid use; MGMT status.
				401 (RT arm/Nordic/NOA)	QLQ-C30	OS	Physical Functioning, Appetite Loss	age; sex; WHO performance status; surgery type; baseline steroid use; MGMT status.
					QLQ-BN20	OS	Future Uncertainity	age; sex; WHO performance status; surgery type; baseline steroid use; MGMT status.
				314 (TMZ or RT + TMZ arm/ Nordic/NOA)	QLQ-C30	OS	Cognitive Functioning	age; sex; WHO performance status; surgery type; baseline steroid use; MGMT status; comorbidity burden.
					QLQ-BN20	OS	Seizures	age; sex; WHO performance status; surgery type; baseline steroid use; MGMT status. comorbidity burden.
				281 (TMZ or RT + TMZ arm/ CE6)	QLQ-C30	OS	Cognitive Functioning, Insomnia	age; sex; WHO performance status; surgery type; baseline steroid use; MGMT status. comorbidity burden.

PRO = patient-reported outcome; OS = overall survival; PFS = progression-free survival; RT = radiotherapy; TMZ = temozolomide; CE6 = Combined European organisation for research and treatment of cancer (EORTC) protocol 6 (chemotherapy-eligible cohort); NOA = Neuro-Oncology working group of the German cancer society (study cohort); QLQ-C30 = European organisation for research and treatment of cancer quality of life Questionnaire–Core 30; QLQ-BN20 = European organisation for research and treatment of cancer brain neoplasm Module; EU = European Union; MMSE = Mini-Mental state Examination. RPA = Recursive partitioning Analysis, MGMT = O⁶-methylguanine–DNA methyltransferase. ^$^Clinical covariates were forced into multivariable models, while PRO/NCB variables were selected using Stepwise procedures. RPA (Recursive partitioning Anssalysis) is a composite prognostic classification incorporating age, performance status, extent of resection, and neurologic function

Seven studies reported multivariate outcomes using the EORTC QLQ-C30^13–18,20^ tool, and four reported using the EORTC QLQ-BN20^14,15,17,19^. Among the studies using the EORTC QLQ-C30 tool to assess OS, four studies included and reported cognitive functioning [14, 15, 17, 18] in multivariable analysis, three included and reported physical functioning [13, 14, 20], and two included and reported social functioning [14, 17]. Role functioning [15], emotional functioning [16], global health status [17], insomnia [14], appetite loss, ^13^ and nausea and vomiting [14] were each included and reported in one study. Three studies reported future uncertainty [14, 16, 19], while motor dysfunction [15], communication deficit [16], visual disorder [14], seizures [14], and weakness in legs [16] were reported in a single study each using the BN-20 tool. Using EORTC QLQ-C30, three studies reported an association between PFS across six domains [13, 15, 20].

Within the included studies, hazard ratios for OS and PFS were obtained from multivariable Cox proportional hazards models, which adjusted for established clinical and tumor-related prognostic factors. Although the specific covariates varied among studies, commonly adjusted variables comprised age, sex, baseline performance status (e.g., WHO or KPS), extent of resection, tumor grade, molecular markers (e.g., IDH mutation and MGMT promoter methylation status), and treatment modality (Table 1).

Most studies evaluated the complete set of domains within the EORTC QLQ-C30 and, where pertinent, the QLQ-BN20; however, only a subset of these domains was incorporated or retained in multivariable models. The selection of PRO domains varied across studies and was founded on prior clinical relevance, univariable screening for survival associations, or the exclusion of correlated domains to reduce collinearity and overfitting. Notably, no study concurrently included all QLQ-C30 or QLQ-BN20 domains within a single multivariable model. This variability in covariate adjustment and PRO domain selection plausibly contributes to the heterogeneity observed between studies in the pooled estimates.

### Overall survival

A summary of meta-analysis results is provided in Fig. 2. Overall pooled analysis of cognitive functioning (HR = 0.94, 95% CI [0.91–0.97]; *I*^*2*^ = 91.5%) (Fig. 2a) and physical functioning (HR = 0.97, 95% CI [0.94-1.0]; *I*^*2*^ = 77.1%) (Fig. 2b) showed to be significantly associated with improved OS, where as social functioning showed no association with OS (HR = 1.02, 95% CI [0.43–2.44]; *I*^*2*^ = 94.5%) (Fig. 2c). When all the functioning scales with at least two studies reporting were pooled, better functioning was associated with improved OS (HR = 0.96, 95% CI [0.93-1.0]; *I*^*2*^ = 88.1%) (Fig. 2d).

A pooled analysis of EORTC QLQ-BN20 showed no significant association between future uncertainty and OS (HR = 1.01, 95% CI [0.94–1.09]; *I*^*2*^ = 85.6%) (Fig. 3).

### Progression-free survival

Pooled analysis of studies reporting Physical functioning showed a significant association with improved PFS (HR = 0.99, 95% CI [0.97-1.0]; *I*^*2*^ = 27.2%) (Fig. 4a) and pooled analysis of all functional scales showed the same direction with improved PFS (HR = 0.99, 95% CI [0.99–0.99]; *I*^*2*^ = 98.9%) (Fig. 4b).

Multiple domain-specific pooled analyses exhibited significant heterogeneity, indicative of variations in patient populations, covariate adjustment methodologies, and analytical techniques across different studies. In areas with a restricted number of contributing studies or consistently high heterogeneity, quantitative synthesis was approached with caution, prioritizing the consistency and directional trends of effects across studies over solely relying on pooled point estimates.

### Risk of bias and certainty assessment

Risk of bias across the eight included randomized trials was generally low, with most studies demonstrating adequate randomization, appropriate outcome measurement, and minimal deviations from intended interventions. The main concerns related to missing PRO data stem primarily from clinical decline in glioma populations and selective reporting of PRO domains, leading to an overall judgment of some concerns but no studies at high risk (Supplementary Fig. 1).

Using the GRADE framework, the certainty of evidence supporting associations between baseline PRO domains and overall survival was rated as moderate. Downgrading was driven primarily by inconsistency, reflected by variability in effect estimates and domain-specific associations across studies, and imprecision, due to wide confidence intervals and a limited number of events for certain PRO domains. Suspected publication bias was considered based on selective reporting of PRO domains and the absence of small trials reporting null effects. Certainty of evidence for progression-free survival was rated as *low*, reflecting fewer evaluable trials, greater between-study heterogeneity, and reduced precision of pooled estimates. Study-level contributors to these judgments, including heterogeneity in PRO domain selection and multivariable adjustment strategies, are summarized in Supplementary Table 3.

## Discussion

In this systematic review and meta-analysis of randomized controlled trials involving 6,846 patients with diffuse gliomas, we found that baseline patient-reported outcomes, particularly cognitive and physical functioning measured using the EORTC QLQ-C30, were independent prognostic indicators of overall survival.

Domain-specific analyses showed that higher cognitive and physical functioning were consistently associated with improved survival. In contrast, social functioning and EORTC QLQ BN20 symptom domains showed no association with our assessed outcomes. These results underscore the importance of implementing functional and cognitive health into the global QoL scores to work as early markers of clinically meaningful outcomes. They may also validate the growing recognition that PROs capture elements of disease burden not detected by objective clinical, radiographic or molecular markers alone.

Our findings align with prior evidence showing that patient reported as well as objective symptoms, neurocognitive decline, and functional impairment often precede radiographic progression in gliomas and may reflect early tumor infiltration, network disruption, and global neural dysfunction [8, 12, 15, 23–26]. Cognitive and physical patient reported functioning emerged as the strongest PRO predictors of survival, which aligns well with current understanding of glioma pathophysiology [17, 27–29]. For instance, cognitive functioning, has been shown to correlate strongly with lesion location, tumor volume, white-matter tract involvement, and molecular subtype factors that directly influence prognosis [30–36] .

Importantly, these domains reflect both neurological integrity and the patient’s ability to maintain daily functioning, serving as integrated markers of tumor behavior and host resilience [37, 38]. Our results extend previous multi-cancer findings by Quinten et al. and Coomans et al. and confirm that the prognostic relevance of PROs is not restricted to broad oncology populations but may also strongly apply to the neuro-oncological patient [15, 18].

These results also support and strengthen recent efforts by the RANO and RANO-PRO working groups that incorporate patient-reported outcomes into glioma trial design and clinical decision-making [1, 39–41]. Traditionally, radiographic markers such as contrast enhancement are increasingly recognized as imperfect surrogates for clinical status due to pseudoprogression, pseudoresponse, steroid effects, as well as interobserver variability [42–47]. The observation that functional and cognitive decline predict survival independent of imaging supports a more multidimensional approach to response assessment in glioma. While our findings do not establish PROs as replacements for imaging-based response criteria, they underscore the potential value of incorporating PRO trajectories alongside radiographic and molecular markers in future research endeavors. Such integrated methodologies may facilitate the refinement of prognostic stratification, improve the interpretation of treatment effects, and augment endpoint sensitivity in clinical trials, subject to direct comparative validation.

### Limitations

This study leverages only high-quality randomized data from published RCTs and applies rigorous methods to isolate the prognostic value of PROs. However, several limitations warrant consideration. Although the majority of included trials utilized both the EORTC QLQ-C30 and QLQ-BN20 instruments, heterogeneity in Patient-Reported Outcome (PRO) reporting emerged at the domain and analytic levels rather than at the instrument level. Specifically, studies varied in the selection of PRO domains incorporated into multivariable models, in whether these domains were chosen a priori or identified through statistical screening, and in whether PROs were analyzed individually or within composite or stepwise frameworks. Consequently, only a subset of studies contributed data to each domain-specific meta-analysis, which limited the precision of the findings and contributed to heterogeneity across studies.

Furthermore, variability in statistical adjustments across trials likely affected the pooled effect estimates. Although all studies controlled for established prognostic factors, the specific covariates and modeling strategies differed: some adjusted for composite indices such as RPA (Recursive Partitioning Analysis) class, others for molecular markers such as MGMT promoter methylation, and still others used trial-level stratification or stepwise selection, as summarized in Table 1. These differences hindered full harmonization of adjusted hazard ratios and may have contributed to residual heterogeneity.

Most studies reported baseline PROs only, preventing evaluation of longitudinal trajectories which may be more predictive of tumor progression and survival than single time-point measurements. Furthermore, analyses were not restricted to specific brain tumor pathologies, which may obscure histology-specific PRO-survival associations that could emerge in larger, more granular datasets. Future research should integrate serial PRO monitoring with imaging, digital neurocognitive testing, and molecular biomarkers to develop hybrid prognostic models. Large collaborative datasets and standardized PRO methodology endorsed by RANO-PRO will be essential for validating PRO-informed survival models. Ultimately, our findings reinforce the need to embed PRO measurement as a routine component of neuro-oncology care and clinical trials, not merely as a supportive endpoint but as a clinically meaningful predictor of patient outcomes.

## Conclusion

This review shows that patient-reported outcomes, especially cognitive and physical functioning, offer independent prognostic value for overall survival in diffuse glioma patients. These domains reflect how patient reported neurological and functional status may not fully be captured by radiographic or molecular markers. While global QoL and most EORTC QLQ BN20 symptom scales lacked consistent prognostic power, the strong links for patient reported cognitive and physical functioning support their routine clinical use and future trial endpoints. As the field advances toward patient-centered response frameworks, adding PROs to RANO criteria and molecular profiling could improve risk stratification, early detection of decline, and personalized treatment. Standardizing and prospectively evaluating PROs in future studies may be vital to enhancing their role in prognosis and therapeutic decision-making.

## Supplementary Information

Below is the link to the electronic supplementary material.

Supplementary Material 1

## Data Availability

The datasets generated during and/or analysed during the current study are available from the corresponding author on reasonable request.
