# Supplementary material for: Patient-reported outcomes as prognostic indicators for overall survival and progression-free survival in brain tumor patients – a systematic review and meta-analysis of randomized clinical trials
Source: J Neurooncol. 2026 Jan 5;176(2):154. doi: 10.1007/s11060-025-05419-w (PMC12769703; doi:10.1007/s11060-025-05419-w)
Supplement: Supplementary file 1 — Supplementary Material 1 [file 11060_2025_5419_MOESM1_ESM.docx]

**Table 1.**

| Database | Search terms | Results |
| --- | --- | --- |
| PUBMED/MEDLINE | (("Brain Neoplasms"[Mesh] OR glioma*[tiab] OR glioblastoma[tiab] OR GBM [tiab] OR "high-grade glioma"[tiab] OR HGG [tiab] OR astrocytoma[tiab] OR oligodendroglioma[tiab]))  AND  (("Quality of Life"[Mesh] OR "Patient Reported Outcome Measures"[Mesh] OR "Patient Reported Outcome*"[tiab] OR PRO[tiab] OR PROM*[tiab] OR "health-related quality of life"[tiab] OR HRQoL[tiab]  OR "EORTC QLQ-C30"[tiab] OR "QLQ-C30"[tiab] OR "QLQ-BN20"[tiab] OR "BN20"[tiab]  OR "FACT-Br"[tiab] OR "FACT G"[tiab] OR "FACT-G"[tiab] OR "MDASI-BT"[tiab] OR "MDASI"[tiab]  OR "PROMIS"[tiab] OR "EQ-5D"[tiab]))  AND  ( radiotherapy*[tiab] OR chemoradiation[tiab] OR chemoradiotherapy[tiab] OR "Stupp protocol"[tiab] OR temozolomide[tiab] OR TMZ [tiab])  AND  (randomized controlled trial[pt] OR randomized[tiab] OR randomized [tiab] OR "clinical trial"[tiab])  NOT (pediatric*[tiab] OR child [Mesh]) | 546 |
| EMBASE | ('brain tumor'/exp OR 'glioma'/exp OR 'glioblastoma'/exp OR 'astrocytoma'/exp OR 'oligodendroglioma'/exp OR 'brain metastasis'/exp OR 'intracranial metastasis'/exp OR  (glioma* OR glioblastoma OR gbm OR high-grade glioma OR hgg OR brain) AND metast*)  AND  (eortc qlq-c30 OR qlq-c30 OR qlq-bn20 OR bn20 OR fact-br OR fact g OR promis OR eq-5d  OR mdasi OR mdasi-bt)  AND  (Radiotherapy OR chemoradiation OR temozolomide OR stereotactic radiosurgery OR  whole brain radiotherapy OR wbrt OR srs)  AND  (randomized OR randomized OR trial OR phase ii OR phase iii OR prospective)  AND  [english]/lim AND [humans]/lim AND [1990–2025]/py  NOT ('child'/exp) | 446 |
| Scopus | (EORTC QLQ-C30 OR QLQ-C30 OR QLQ-BN20 OR FACT-Br OR MDASI OR PROMIS OR EQ-5D)  AND  (glioma* OR glioblastoma OR GBM OR high-grade glioma OR HGG  OR brain metast* OR intracranial metast* OR meningioma*)  AND  (radiotherap* OR chemoradiation OR temozolomide OR TMZ  OR stereotactic radiosurgery OR SRS OR whole brain radiotherap*  OR WBRT OR gamma knife OR cyberknife)  AND  (random* OR trial OR phase II OR phase III OR prospective) | 147 |

**Supplementary Table 2. Inclusion and exclusion criteria for study eligibility.**

| Inclusion Criteria | Exclusion Criteria |
| --- | --- |
| • Randomized phase II-IV clinical trials. | • Retrospective studies, case series, case reports, reviews, or abstracts only. |
| • Adult patients (≥18 years). | • Pediatric populations or mixed cohorts without separable adult data. |
| • High-grade gliomas (glioblastoma, anaplastic astrocytoma, anaplastic oligodendroglioma). | • Benign tumors or non-glioma CNS tumors without glioma-specific data. |
| • Anti-tumor treatments: radiotherapy, chemoradiation, temozolomide, SRS, WBRT, or systemic/locoregional therapies. | • Studies of surgery alone or supportive-care interventions without oncologic treatment comparison. |
| • Validated PRO instruments used (QLQ-C30 and QLQ-BN20). | • Non-validated PRO tools or clinician-reported scores only (e.g., KPS/ECOG). |
| • PROs assessed in relation to survival (OS and/or PFS) in uni- or multivariable models. | • PROs reported only descriptively without any survival analysis. |
| • Full-text available in English with extractable survival effect estimates. | • Insufficient data for extraction or duplicate reports without added information. |
| • Published 2000 onwards. | • Preclinical, animal, or in-vitro studies. |

**Supplementary Table 3. Certainty of evidence of the included studies**

| **Outcome** | **Study design** | **Risk of bias** | **Inconsistency** | **Indirectness** | **Imprecision** | **Publication bias** | **Overall certainty** | **Key reasons for downgrading** |
| --- | --- | --- | --- | --- | --- | --- | --- | --- |
| Overall survival | Randomized controlled trials | Not serious | Serious | Not serious | Serious | Suspected | Moderate | Between-study heterogeneity in domain-specific effects; wide confidence intervals for several PRO domains; selective reporting of PRO scales |
| Progression-free survival | Randomized controlled trials | Not serious | Serious | Not serious | Serious | Suspected | Low | Limited number of trials; substantial heterogeneity; imprecise estimates with wide confidence intervals; selective PRO reporting |

**Supplementary Figure 1. Risk of Bias Assessment using Risk of Bias tool for Randomized trials.**
